# Oral-Mucosal PCO_2_ during hemorrhagic shock closely Monitors its time Course, Severity, and reversal outperforming blood lactate measurement

**DOI:** 10.1016/j.resplu.2024.100814

**Published:** 2024-11-28

**Authors:** Armin Razi, Iyad M. Ayoub, Alvin Baetiong, Salvatore Aiello, Moaz Bin Saeed, Martin Pelletier, Cara Joyce, Raúl J. Gazmuri

**Affiliations:** a Resuscitation Institute at Rosalind Franklin University of Medicine and Science, North Chicago, IL, USA; b Critical Care Section at the CAPT James A. Lovell Federal Health Care Center, North Chicago, IL, USA; c Resuscitation Therapeutics, North Chicago, IL, USA; d Loyola University Chicago Health Sciences Campus, Maywood, IL, USA

**Keywords:** Hemorrhagic shock, Capnometry, Swine, P_OM_CO_2_, Lactate

## Abstract

**Introduction:**

Given the redistribution of blood flow away from non-immediately vital territories during hemorrhagic shock, we investigate whether monitoring the oral mucosal PCO_2_ (P_OM_CO_2_) as a surrogate of splanchnic circulation, could closely recognize the onset, assess severity, and identify reversal of hemorrhagic shock.

**Material and methods:**

The study was performed on six male pigs (38.4 ± 1.6 kg). P_OM_CO_2_ was measured using a non-invasive sensor clipped to the cheek. Blood was removed over 120 min from the right atrium modeling spontaneous bleeding and reinfused in 20 min observing the animal for 180 min.

**Results:**

A total of 1485 ± 188 ml (i.e., 64.6 ± 9.5 % of the estimated blood volume) was removed inducing severe hemorrhagic shock. P_OM_CO_2_ closely paralleled the blood volume change (R^2^ = 0.59, p < 0.001) showing an early and steady increase from 86 ± 5 mmHg at baseline to 152 ± 28 mmHg after 120 min. Blood reinfusion reduced the P_OM_CO_2_ to 138 ± 37 mmHg after 15 min and 97 ± 34 mmHg at the end of 180 min, coincident with the reversal of hemorrhagic shock. Blood lactate less accurately paralleled the blood volume change (R^2^ = 0.14, p < 0.001) showing a slower increase during hemorrhagic shock (from 1.1 ± 0.3 to 4.2 ± 1.8 mmol/l after 120 min) with further increase to 5.2 ± 1.7 mmol/l following blood reinfusion at minute 150 min, remaining at 4.0 ± 1.5 mmol/l by the end of the 180-minute observation period.

**Conclusions:**

P_OM_CO_2_ monitoring may provide a clinically practical non-invasive indicator of hemorrhagic shock assessing its severity, clinical course, and treatment effect outperforming blood lactate which exhibited a slower and delayed response.

## Introduction

Hemorrhagic shock is a life-threatening emergency characterized by a significant decrease in blood volume leading to reduction in tissue oxygen delivery. [1] Prompt recognition, assessment of severity, and targeted treatment are critical to survival of the affected individual. One key feature of hemorrhagic shock and other low flow states is the redistribution of blood flow away from non-immediately vital territories such as the skin, skeletal muscle, and the splanchnic territory to preferentially perfuse vital organs. [2] However, a sustained reduction in blood flow to these non-immediately vital territories leads to tissue hypoxia and a cascade of adverse events that if not promptly recognized and treated risk development of multiorgan system failure and macrocirculatory dysfunction despite restoration of intravascular blood volume. [3] .

In contrast to assessing the macrocirculation, assessing the microcirculation is challenging and requires monitoring changes in specific tissue beds. [4] Increased partial pressure of carbon dioxide (PCO_2_) at the tissue level is a useful surrogate of decreased microcirculatory blood flow and indicator of organ ischemia that can be used to recognize and assess the severity of circulatory shock. 5, 6, 7, 8.

**Fig. 1:**
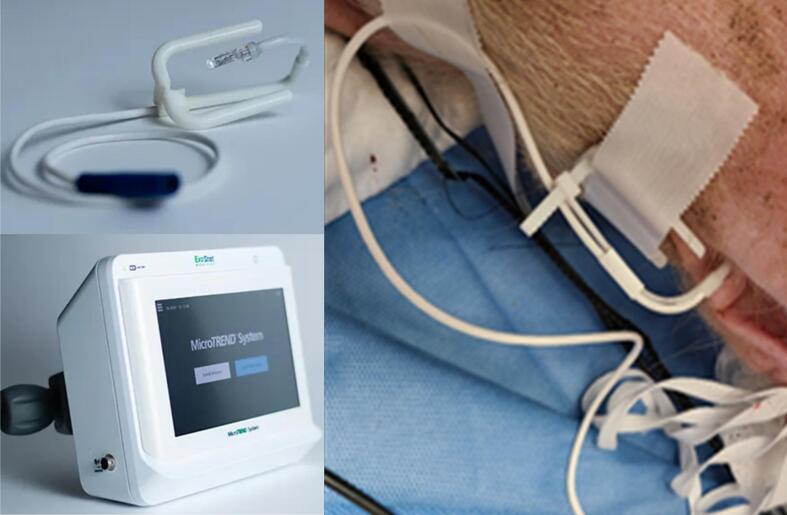
MicroTrend™ System showing the sensor clipped to the animal’s cheek and its monitoring device, which was connected to our LabVIEW system.

Previous studies showing that gastric wall PCO_2_ can be used to assess microcirculatory changes in low-flow states, including hemorrhagic shock, [9] led to the clinical development of gastric tonometry. [10] However, gastric tonometry is semi-invasive, intermittent, and prone to errors related to acidic gastric fluid and equilibration delays necessitating acid suppression to minimize inaccuracies. Additional studies suggested that esophageal wall PCO_2_ may serve to assess the microcirculation during circulatory shock, [11] leading investigators to explore sublingual measurements as a clinically more practical method. Initial studies were conducted in rats showing a correlation between sublingual tissue PCO_2_ and gastric PCO_2_ measurements along with concurrent changes in cardiac index, mean aortic pressure, end-tidal PCO_2,_ and arterial lactate. [12] Subsequent studies confirmed these associations offering a clinically more practical alternative to gastric tonometry during hemorrhagic and septic shock. 10, 12 More recently, ExoStat Medical (Prior Lake, MN) developed a technology to continuously monitor the oral mucosal PCO_2_ (P_OM_CO_2_) as a surrogate of splanchnic perfusion known as MicroTrend™ System. [13] The P_OM_CO_2_ is measured non-invasively through a disposable PCO_2_ sensor clipped to the cheek (Fig. 1). In a swine model of coagulopathic junctional hemorrhage the MicroTrend™ System demonstrated an inverse correlation between blood pressure and P_OM_CO_2_. [13].

The current study was designed to assess the MicroTrend™ System in a swine model of hemorrhagic shock, modeling spontaneous bleeding over 120 min and reversal by blood reinfusion over 20 min while monitoring multiple hemodynamic and metabolic variables aimed at assessing the ability of P_OM_CO_2_ to accurately monitor the onset of hemorrhagic shock, assess its severity, and identify its reversal.

## Material and methods

The study was approved by the Institutional Animal Care and Use Committee at Rosalind Franklin University of Medicine and Science, North Chicago, IL. The experiments were acute without recovery from anesthesia using a swine model of controlled hemorrhagic shock developed at the Resuscitation Institute. [14].

### Animal Housing and Husbandry

The experiments were conducted in six Yorkshire x Landrace castrated male pigs weighing 36.7 to 40.3 kg purchased from Oak Hill Genetics, Ewing, IL. The animals were group-housed in pens in the Biological Resource Facility (AAALAC accredited facility) at Rosalind Franklin University of Medicine and Science. Lights were set at the recommended illumination levels of a 12/12-hour cycle controlled via automatic timers. The temperature was maintained between 61F and 81F. Assessment for general health and well-being was performed daily by animal care technicians and veterinarians and by investigators the day before each experiment.

### Animal preparation

The animals were fasted overnight, sedated with ketamine hydrochloride (Dechra Vet, Leawood, KS), (30 mg·kg^−1^ intramuscularly), anesthetized with propofol (Zoetis, Parsipanny, NJ) (2 mg·kg^−1^ intravenous through an ear vein), intubated with a 7.5 mm endotracheal tube, and started on isoflurane (Pivetal®) anesthesia (1.5 % to 4.0 %) titrated to sustain a surgical plane. Positive pressure ventilation was provided with 50 % oxygen using a volume-controlled ventilator (840 Ventilator System, Nellcor Puritan Bennett) set to deliver a tidal volume of 10 ml·kg^−1^ and peak flow of 60 l·min^−1^. The respiratory rate was adjusted to initially maintain an end expired PCO_2_ (P_ET_CO_2_) between 35 and 45 mmHg (Capnogard, Novometrix Medical Systems).

**Fig. 2:**
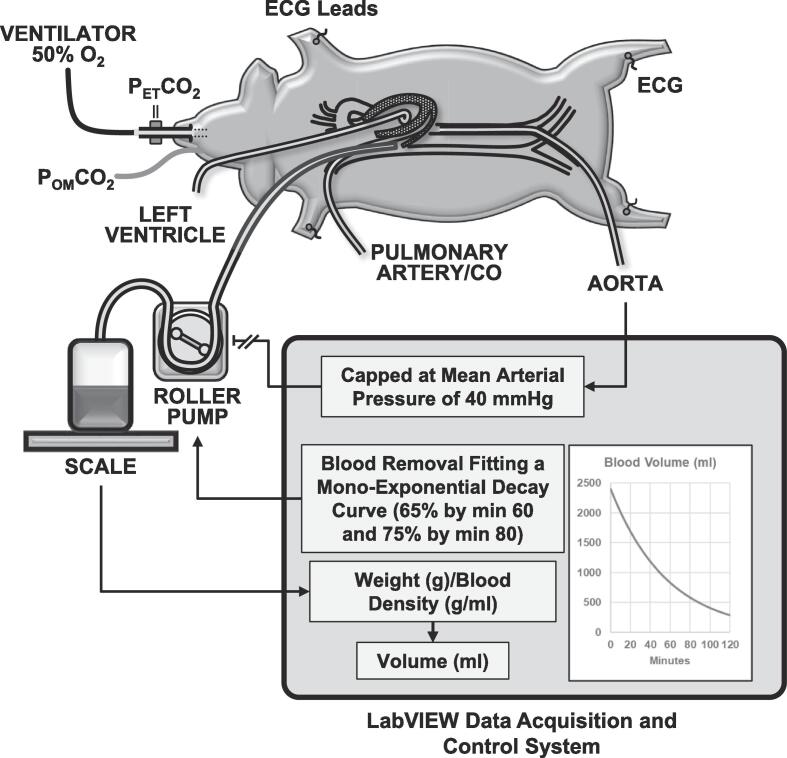
Swine model of hemorrhagic shock depicting blood removal from the right atrium using a LabVIEW-controlled roller pump following a mono-exponential decay function to remove 65 % of the animal blood volume (i.e., 60 ml/kg) by minute 60 and 75 % by minute 80 stopping the pump if the mean arterial pressure decreased below 40 mmHg. The blood volume was determined by continuously measuring the blood weight in an electronic scale converting to volume based on blood density (1.06 g/ml). P_ET_CO_2_ = End-tidal PCO_2_; P_OM_CO_2_ = Oral mucosal PCO_2_; ECG = Electrocardiogram; CO = Cardiac output.

Animals were then instrumented by advancing (1) a 6-Fr fluid-filled catheter from the right femoral artery into the descending thoracic aorta for blood pressure monitoring; (2) a 7-Fr balloon-tipped thermodilution pulmonary artery catheter from the right internal jugular vein into the pulmonary artery for mixed venous (MV) blood sampling, core temperature measurement, and cardiac output by bolus thermodilution (Edwards Vigilance II, Irvine, CA); (3) a 6-Fr fluid-filled pigtail catheter from the right carotid artery into the left ventricle for measuring left ventricular pressures and blood sampling; and (4) a 14-Fr cannula advanced through the right external jugular vein into the right atrium for blood removal using cut-down technique (Fig. 2). Core temperature was maintained between 37.5 °C and 38.5 °C with a water-circulated blanket during the surgery (Blanketrol II, Cincinnati SubZero).

### P_OM_CO_2_ monitoring

The P_OM_CO_2_ sensor was calibrated with two known CO_2_ concentrations and placed in the animal’s right cheek. The P_OM_CO_2_ data was displayed on the MicroTrend™ monitor and simultaneously displayed and recorded using a custom-developed LabVIEW data acquisition and analysis system.

### ***Hemorrhagic shock*** model ***of spontaneous bleeding***

A closed-loop system modeling spontaneous bleeding previously developed in LabVIEW 6.0 at the Resuscitation Institute was used for the experiments. [14] Briefly, blood was removed using a roller pump from the right atrium into a 2,000 ml blood transfer bag containing 10 U/ml heparin based on the anticipated blood volume to be removed. The transfer bag was placed on an electronic scale enabling continuous gravimetric measurement of blood volume (blood density = 1.06 g/ml). The blood was removed according to a mono-exponential decay function to model spontaneous bleeding, i.e., removing a maximum of 60 % of the estimated swine blood volume (60 ml/kg) by minute 60 and 75 % by minute 80, capping the blood removal if the mean arterial pressure decreased below 40 mmHg as shown in Fig. 2. The removed blood was reinfused over 20 min after 120 min from the start of blood removal and the animals observed for an additional 40 min for a total of 180 min from the start of blood removal. The animals were euthanized at the end of the experiment by injection of KCl (150 mg/kg injection) in the right atrium.

### Measurements

**Fig. 3:**
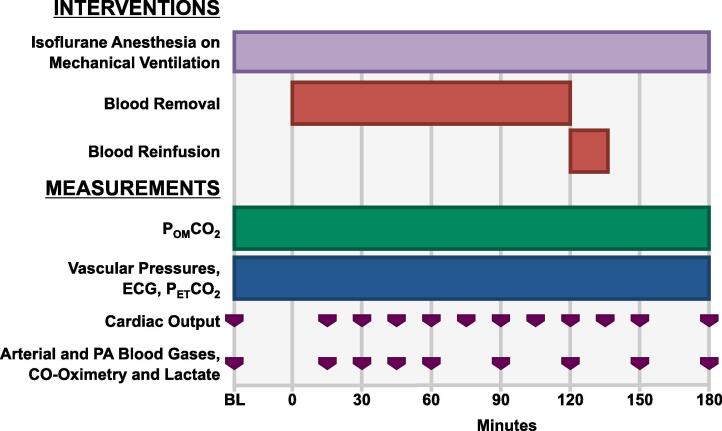
Experiment protocol. P_OM_CO_2_ = Oral mucosal PCO_2_; ECG = Electrocardiogram; P_ET_CO_2_ = End-tidal PCO_2_; PA = Pulmonary artery.

The timeline of hemodynamic and metabolic measurements is shown in Fig. 3. Cardiac output was measured by bolus thermodilution in duplicate after injection of 0.9 % NaCl (10 ml) into the right atrium, with a third measurement performed if the difference between the first and second exceeded 10 %. The averaged cardiac output was indexed to body surface area using the Kelley equation (body surface area [m^2^] = 0.073·body-weight^2/3^ [kg]).[15] Vascular pressures were calibrated using fluid-filled systems and a digital pressure gauge (DPG1000, Omega Engineering) zeroed to mid-cavity level. All signals were sampled and digitized at 250 Hz using a 16-bit data acquisition board (AT-MIO-16XE-50; National Instruments) and analyzed using custom-developed software (LabVIEW 6.0, National Instruments).

The left and right ventricular stroke work index (LVSWI and RVSWI) were determined by multiplying the stroke volume index by the difference between systolic and end-diastolic left ventricular pressures and between the systolic pulmonary artery pressure and the mean right atrial pressure, respectively, and expressed in centijoules (cJ) by multiplying by 0.0133. [16] The systemic vascular resistance (SVR) was calculated from the difference between mean aortic and mean right atrial pressure divided by cardiac output and reported in dynes·s/cm^−5^.

Blood was sampled from the left ventricle (arterial blood) and the pulmonary artery (mixed venous blood) and processed on-site for pH, PO_2_, PCO_2_, hemoglobin, and lactate using a cartridge-based device (OPTI CCA-TS Blood Gas and Electrolyte Analyzer, OPTI Medical Systems) and for common hemoglobin types (oxy-, met-, carboxy-, and reduced-) using a co-oximeter (AVOXimeter 4000, AVOX Systems Inc., San Antonio, TX). The O_2_ content in the arterial blood and pulmonary artery (mixed venous) was calculated according to:

O_2_ content (ml/dl) = hemoglobin (g/dl) × 1.39 (ml/g) × SFO_2_ + 0.003 (ml/dl × mmHg^−1^) × PO_2_ (mmHg).

where 1.39 denotes ml of O_2_ bound to 1 g of hemoglobin (Hufner’s number), SFO_2_ is the fraction of oxyhemoglobin relative to the four hemoglobin types, and 0.003 is the O_2_ solubility coefficient. Systemic oxygen delivery and consumption were calculated from arterial O_2_ content multiplied by cardiac index and from the difference between arterial and pulmonary artery (mixed venous) O_2_ content multiplied by cardiac index and reported in ml/min·m^−2^.

### Statistical analysis

**Fig. 4:**
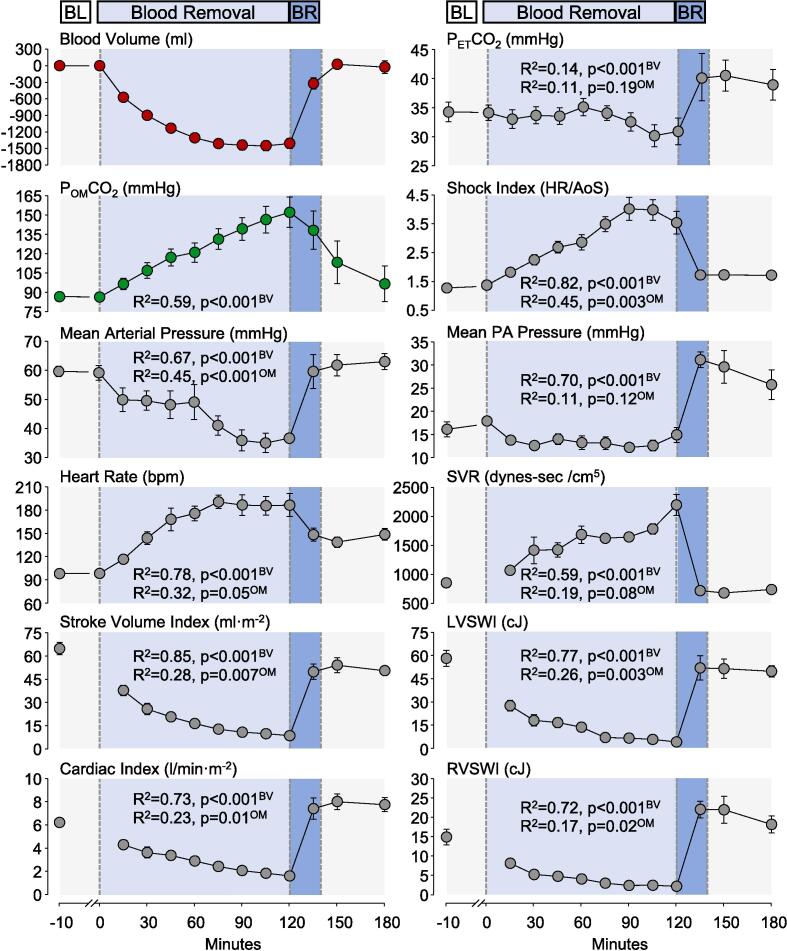
Effects of blood volume changed during hemorrhagic shock and its reversal on P_OM_CO_2_ and hemodynamic variables. BL = Baseline; BR = Blood Reinfusion; P_ET_CO_2_ = End tidal PCO_2_; HR = Heart Rate; AoS = Aortic Systolic Pressure; PA = Pulmonary Artery; SVR = Systemic Vascular Resistance; LVSWI = Left Ventricular Stroke Work Index; RVSWI = Right Ventricular Stroke Work Index. Data are shown as mean ± SEM with the R^2^ and corresponding p-values shown for the regression of blood volume (BV) and P_OM_CO_2_ (OM) on the shown variables.

**Fig. 5:**
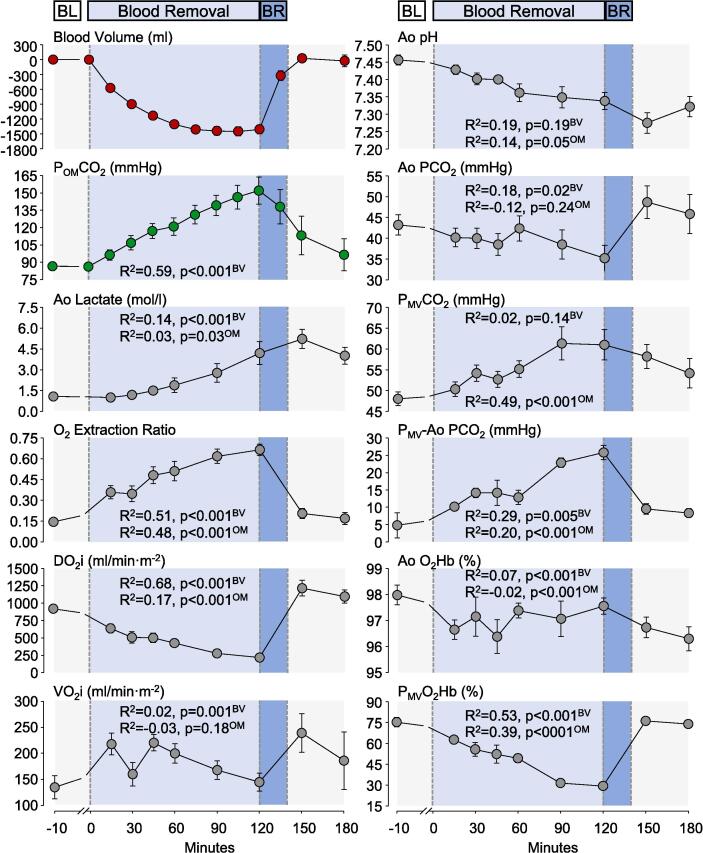
Effects of blood volume changed during hemorrhagic shock and its reversal on P_OM_CO_2_ and metabolic variables. BL = Baseline; BR = Blood Reinfusion; Ao = Aorta; DO_2_i = Systemic Oxygen Delivery index; VO_2_i = Systemic Oxygen Consumption index; MV = Mixed Venous; O_2_Hb = Oxyhemoglobin. Data are shown as mean ± SEM with the R^2^ and corresponding p-values for the regression of blood volume (BV) and P_OM_CO_2_ (OM) on the shown variables.

We assessed the time-coincident associations between the removed blood volume, P_OM_CO_2_, and hemodynamic and metabolic parameters using generalized estimating equations (GEE) models. The first set of GEE models regressed the removed blood volume on P_OM_CO_2_ and each hemodynamic and metabolic parameter to determine which measures tracked closely the blood removal and reinfusion. A second set of GEE models regressed the P_OM_CO_2_ on the hemodynamic and metabolic parameters to determine which measurements were best tracked by the P_OM_CO_2_. For each model, the within-animal correlation of the dependent variable over time was addressed by specifying an autoregressive correlation structure reporting the R-squared (R^2^) for each model calculated as described by Zheng. [17] The R^2^ is a measure of model fit, where higher values represent stronger associations across animals and time points. R^2^ are reported with their p-values in Fig. 4, Fig. 5. In addition, beta coefficients with their 95 % confidence intervals representing the estimated mean change in the dependent variable for a unit change in the explanatory variable, similar to simple slopes, are reported along with number of observations in a Supplement. The data are presented as mean ± SEM in the graphs and as mean ± SD in the text.

The sample size of six experiments was considered adequate given the high precision with which the blood was removed, using a roller pump controlled by a close-loop system modeling a monoexponential decay function, that resulted in a small SEM for each of the 11 blood volume measurements, successfully demonstrating a statistically significant association with the primary endpoint (i.e., P_OM_CO_2_) and several secondary endpoints.

## Results

All six pigs survived until the end of the experiment. The hemodynamic and metabolic data are shown in Fig. 4, Fig. 5, with the change in blood volume and P_OM_CO_2_ graphs shown in both figures for reference. A total of 1,485 ± 188 ml corresponding to 64.6 ± 9.5 % of the estimated blood volume was removed promoting a state of severe hemorrhagic shock, accompanied by its characteristic hemodynamic manifestations including decrease in arterial blood pressure, stroke volume index, cardiac index, left and right ventricular stroke index and increase in heart rate, shock index, and systemic vascular resistance (Fig. 4). These hemodynamic effects were accompanied by corresponding metabolic manifestations including decrease in systemic oxygen delivery with marked increase in systemic oxygen extraction while preserving systemic oxygen consumption (Fig. 5). There was also a progressive widening of the P_MV_-Ao PCO_2_ gradient resulting from a prominent increase in the P_MV_CO_2_ with minor decrease in arterial PCO_2_, the latter accompanied by a decrease in end-tidal PCO_2_, arterial pH, increased in blood lactate and decrease in P_MV_O_2_Hb saturation (Fig. 5). A total of 1,339 ± 253 ml of blood was reinfused over 20 min after minute 120, corresponding to 89.6 ± 8.8 % of the blood removed. Blood reinfusion was accompanied by rapid reversal of these hemodynamic and metabolic abnormalities except for blood lactate (as noted below) and a transient increase in P_ET_CO_2._

The P_OM_CO_2_ closely paralleled the time course of blood volume changes during hemorrhagic shock (R^2^ = 0.59, p < 0.001) showing an early and sustained increase from 86 ± 5 mmHg at baseline to 152 ± 28 mmHg after 120 min. Blood reinfusion rapidly reduced the P_OM_CO_2_ to 138 ± 37 mmHg after 15 min and 97 ± 34 mmHg at the end of 180 min, coincident with reversal of hemorrhagic shock. Blood lactate less accurately paralleled the time course of blood volume change (R^2^ = 0.14, p < 0.001) showing an increase during hemorrhagic shock (from 1.1 ± 0.3 to 4.2 ± 1.8 mmol/l after 120 min) with further increase (not decrease) to 5.2 ± 1.7 mmol/l following blood reinfusion at minute 150 min, remaining at 4.0 ± 1.5 mmol/l by the end of the 180-minute observation period.

## Discussion

The study demonstrates the ability of P_OM_CO_2_ to non-invasively and timely track hemodynamic and metabolic changes occurring during hemorrhagic shock and its reversal.

### ***Tissue*** PCO_2_***: An early sensitive Sign of ischemia***

Elevation in tissue PCO_2_ during low-flow states results from two distinct sequential additive processes. Initially, a reduction in tissue blood flow while maintaining oxygen demands (i.e., before development of ischemia) prompts CO_2_ accumulation consequent to reduced clearance of aerobically generated CO_2_. Subsequently, further reduction in blood flow to levels that fail to meet the metabolic tissue demands prompts ischemia with anaerobic H^+^ generation following breakdown of high-energy phosphate compounds such as ATP. The excess of H^+^ is buffered by HCO_3_^–^ producing CO_2_ (i.e., [H^+^] + [HCO_3_^–^] ⇆ [H_2_O] + [CO_2_]) accentuating tissue PCO_2_. Accordingly, an increase in tissue PCO_2_ is an early and sensitive marker of reduced organ blood flow enabling, if detected, to initiate therapeutic interventions even before tissue ischemia develops. 4, 18.

### Preferential ***redistribution of blood flow to vital organs***

Vital organs, including the heart and brain, have robust pressure autoregulation enabling them to maintain blood flow to meet their metabolic needs as blood pressure decreases. In contrast, non-immediately vital organs and territories such as the splanchnic territory lack robust pressure autoregulation and are more prone to developed ischemia as blood pressure decreases. [19] The pressure autoregulation of the oral mucosal is similar to that of the splanchnic territory providing a clinically useful window to monitor the physiological response to reductions in systemic blood flow. Accordingly, the progressive increase in P_OM_CO_2_ observed during hemorrhagic shock is indicative of progressive reduction in splanchnic blood flow consequent to the expected preferential redistribution of blood flow away from the splanchnic territory favoring vital organ perfusion under conditions of reduced intravascular volume.

Although reduction of blood flow to the oral mucosal and the splanchnic territory poses no immediate vital risk, it may serve, as this study demonstrates, to monitor the physiological response to hemorrhagic shock and guide its management. Changes in P_OM_CO_2_ closely followed changes in mean aortic pressure, heart rate, shock index, cardiac index, stroke volume, systemic vascular resistance, left and right ventricular work, systemic oxygen extraction ratio, mixed venous PCO_2_, and the PCO_2_ gradient between arterial and mixed venous PCO_2_. Accordingly, P_OM_CO_2_ non-invasively tracked continuously the hemodynamic changes characteristic of hemorrhagic shock.

### Outperforming ***blood lactate measurements***

Of substantial translational relevance is that P_OM_CO_2_ outperformed blood lactate measurements, timely tracking the changes on blood volume and the corresponding hemodynamic and metabolic effects. Lactate is commonly measured in circulatory shock to aid in the diagnosis, assessment of severity, monitoring responsiveness to treatment, and prognosis; especially in septic shock despite receiving only a “weak recommendation” by the surviving sepsis campaign guidelines. [20] Lactate is a multifunctional signaling molecule influenced by a variety of metabolic process beyond tissue ischemia and also by sympathetic stimulation. 21, 22 In addition, as observed in our study, the lactate increase during hemorrhagic shock is slow [23] and its reversal delayed upon correction of hemorrhagic shock, [13] in part attributed to diminished plasma clearance. [24].

Moreover, blood lactate levels are measured intermittently, e.g., every two to four hours, [25] lagging behind the underlying physiologic event (as observed in our study) precluding timely adjustment of interventions. In the ANDROMEDA-SHOCK clinical trial, guiding treatment base on capillary refill time compared to lactate measurements resulted in less vasopressor therapy, less fluid administration, less organ dysfunction at 72 h, and a robust trend toward lower mortality. [26] However, we believe these measurements are complimentary, with P_OM_CO_2_ directly and continuously monitoring tissue perfusion enabling real-time titration of interventions and blood lactate intermittently assessing the severity of tissue ischemia along with effects that follow including resolution of sympathetic response, decreased catecholamine administration, lactate metabolism, and repayment of oxygen debt.

### Limitations

The findings apply to a highly controlled model of hemorrhagic shock in healthy animals without underlying comorbidities and under anesthesia precluding directly extrapolating the findings to more complex settings often clinically observed in patients. The findings apply to hemorrhagic shock and may not be generalizable to other types of shock and especially to distributive forms of shock characterized by more complex microcirculatory abnormalities dissociated from the macrohemodynamics.

## Conclusions

In conclusion, measuring P_OM_CO_2_ with the MicroTrend™ System may provide a clinically practical means to monitor hemorrhagic shock, assessing its severity, clinical course, treatment effect, and reversal. P_OM_CO_2_ outperformed lactate measurement which exhibited a delayed response, providing a quick real-time measurement by only clipping a sensor to the cheek of a patient.

## CRediT authorship contribution statement

**Armin Razi:** Investigation, Formal analysis, Data curation, Writing – original draft, Visualization. **Iyad M. Ayoub:** Supervision, Validation. **Alvin Baetiong:** Software, Investigation, Data curation. **Salvatore Aiello:** Investigation, Supervision. **Moaz Bin Saeed:** Investigation, Data curation, Writing – original draft, Visualization. **Martin Pelletier:** Investigation. **Cara Joyce:** Formal analysis. **Raúl J. Gazmuri:** Conceptualization, Methodology, Validation, Formal analysis, Data curation, Writing – review & editing, Visualization, Supervision, Project administration, Funding acquisition.

## Declaration of competing interest

The authors declare that they have no known competing financial interests or personal relationships that could have appeared to influence the work reported in this paper.
