# Supplementary material for: Oral-Mucosal PCO2 during hemorrhagic shock closely Monitors its time Course, Severity, and reversal outperforming blood lactate measurement
Source: Resusc Plus. 2024 Nov 28;20:100814. doi: 10.1016/j.resplu.2024.100814 (PMC11629581; doi:10.1016/j.resplu.2024.100814)
Supplement: Supplementary Data 1 [file mmc1.docx]

Table 1: Associations and model fit using generalized estimating equations.

| **Variables** | **N observations** | **Beta coefficient (95% CI)** | **p-value** | **R^2^ with blood volume** |
| --- | --- | --- | --- | --- |
| P_OM_CO_2_ (mmHg) | 72 | -14.3 (-20.9, -7.7) | <0.001 | 0.59 |
| Ao, mean calculated (mmHg) | 72 | 27.0 (18.8, 35.2) | <0.001 | 0.67 |
| HR (LV) (min^-1^) | 72 | -10.9 (-16.2, -5.6) | <0.001 | 0.78 |
| Stroke volume index (ml/beat-m^-2^) | 65 | 25.8 (22.3, 29.3) | <0.001 | 0.85 |
| Cardiac index (L/min-m^-2^) | 65 | 174 (134, 215) | <0.001 | 0.73 |
| P_et_CO_2_ (mmHg) | 72 | 72 (57, 87) | <0.001 | 0.14 |
| Shock Index (HR/AoS) | 72 | -375 (-498, -252) | <0.001 | 0.82 |
| PA, mean calculate (mmHg) | 72 | 47 (34, 59) | <0.001 | 0.70 |
| SVR (dynes-sec/cm^5^) | 65 | -0.55 (-0.83, -0.27) | <0.001 | 0.59 |
| LVSWI (cJ) | 63 | 19.6 (16.1, 23.1) | <0.001 | 0.77 |
| RVSWI (cJ) | 65 | 48 (40, 56) | <0.001 | 0.72 |
| Lactate, Ao (mmol/L) | 47 | 84 (38, 131) | <0.001 | 0.14 |
| VO_2_/DO_2_ (ratio) | 47 | -2245 (-2528, -1962) | <0.001 | 0.51 |
| DO_2_I (ml/min-m^-2^) | 47 | 1.30 (1.13, 1.47) | <0.001 | 0.68 |
| VO_2_I (ml/min-m^-2^) | 47 | 2.35 (0.92, 3.77) | 0.001 | 0.02 |
| pH, Ao (units) | 47 | -1678 (-4166, 810) | 0.19 | 0.11 |
| PCO_2_, Ao (mmHg) | 47 | 27.5 (5.1, 49.9) | 0.02 | 0.18 |
| PCO_2_, PA (mmHg) | 47 | -18.0 (-42.2, 6.1) | 0.14 | 0.02 |
| PA-Ao PCO_2_ | 47 | 27.5 (5.1, 49.9) | 0.005 | 0.29 |
| O_2_Hb, Ao (%) | 47 | -81 (-116, -46) | <0.001 | 0.07 |
| O_2_Hb, PA (%) | 47 | 25.2 (19.1, 31.2) | <0.001 | 0.53 |
| HCO3 (OPTI), Ao (mmol/L) | 47 | 87 (31, 143) | 0.002 | -0.18 |
| BE (OPTI), Ao (mmol/L) | 47 | 82 (40, 124) | <0.001 | -0.34 |
| P_OM_CO_2_ = Oral mucosal PCO_2_; Ao= Aorta; HR = Heart rate; LV = Left ventricle; P_et_CO_2_ = End tidal PCO_2_; AoS = Aortic systolic pressure; PA = Pulmonary artery; SVR = Systemic vascular resistance; LVSWI = Left ventricular stroke work index; RVSWI = Right ventricular stroke work index. VO_2_i = Systemic oxygen consumption; DO_2_i = Systemic oxygen delivery; O_2_Hb = Oxyhemoglobin; HCO3 = Bicarbonate; BE = Base excess. | | | | |

Table 2: Associations and model fit using generalized estimating equations.

| **Variables** | **N observations** | **Beta coefficient (95% CI)** | **p-value** | **R^2^ with P_OM_CO_2_** |
| --- | --- | --- | --- | --- |
| Ao, mean calculate (mmHg) | 72 | -0.61 (-0.96, -0.27) | <0.001 | 0.45 |
| HR (LV) (min^-1^) | 72 | 0.19 (0.00, 0.38) | 0.05 | 0.32 |
| Stroke volume index (ml/beat-m^-2^) | 65 | -0.42 (-0.72, -0.12) | 0.007 | 0.28 |
| Cardiac index (L/min-m^-2^) | 65 | -2.8 (-5.1, -0.6) | 0.01 | 0.23 |
| P_et_CO_2_ (mmHg) | 72 | -0.95 (-2.37, 0.47) | 0.19 | 0.11 |
| Shock index (HR/AoS) | 72 | 7.4 (2.5, 12.3) | 0.003 | 0.45 |
| PA, mean calculate (mmHg) | 72 | -0.59 (-1.34, 0.16) | 0.12 | 0.11 |
| SVR (dynes-sec /cm^5^) | 65 | 0.009 (-0.001, 0.018) | 0.08 | 0.19 |
| LVSWI (cJ) | 63 | -0.27 (-0.45, -0.10) | 0.003 | 0.26 |
| RVSWI (cJ) | 65 | -0.73 (-1.36, -0.10) | 0.02 | 0.17 |
| Lactate, Ao (mmol/L) | 47 | 4.1 (0.4, 7.9) | 0.03 | 0.03 |
| VO_2_/DO_2_ (ratio) | 47 | 87 (59, 116) | <0.001 | 0.48 |
| DO_2_I (ml/min-m^-2^) | 47 | -0.033 (-0.049, -0.018) | <0.001 | 0.17 |
| VO_2_I (ml/min-m^-2^) | 47 | -0.03 (-0.07, 0.01) | 0.18 | -0.03 |
| pH, Ao (units) | 47 | -86 (-173, 0) | 0.05 | 0.14 |
| PCO_2_, Ao (mmHg) | 47 | -0.64 (-1.71, 0.43) | 0.24 | -0.12 |
| PCO_2_, PA (mmHg) | 47 | 2.2 (1.4, 3.1) | <0.001 | 0.49 |
| PA-Ao PCO_2_ | 47 | 1.4 (0.7, 2.1) | <0.001 | 0.20 |
| O_2_Hb, Ao (%) | 47 | 3.4 (2.1, 4.6) | <0.001 | -0.02 |
| O_2_Hb, PA (%) | 47 | -0.82 (-1.14, -0.50) | <0.001 | 0.39 |
| HCO3 (OPTI), Ao (mmol/L) | 47 | 3.4 (2.1, 4.6) | <0.001 | -0.06 |
| BE (OPTI), Ao (mmol/L) | 47 | -0.82 (-1.14, -0.50) | <0.001 | 0.03 |
| P_OM_CO_2_ = Oral mucosal PCO_2_; Ao= Aorta; HR = Heart rate; LV = Left ventricle; P_et_CO_2_ = End tidal PCO_2_; AoS = Aortic systolic pressure; PA = Pulmonary artery; SVR = Systemic vascular resistance; LVSWI = Left ventricular stroke work index; RVSWI = Right ventricular stroke work index. VO_2_i = Systemic oxygen consumption; DO_2_i = Systemic oxygen delivery; O_2_Hb = Oxyhemoglobin; HCO3 = Bicarbonate; BE = Base excess. | | | | |
